# Elevated Serum Uric Acid Is Associated with High Circulating Inflammatory Cytokines in the Population-Based Colaus Study

**DOI:** 10.1371/journal.pone.0019901

**Published:** 2011-05-20

**Authors:** Tanica Lyngdoh, Pedro Marques-Vidal, Fred Paccaud, Martin Preisig, Gérard Waeber, Murielle Bochud, Peter Vollenweider

**Affiliations:** 1 Institute of Social and Preventive Medicine (IUMSP), University of Lausanne, Lausanne, Switzerland; 2 Department of Psychiatry, CHUV, Lausanne, Switzerland; 3 Department of Medicine, Internal Medicine, CHUV, Lausanne, Switzerland; Massachusetts General Hospital and Harvard University, United States of America

## Abstract

**Background:**

The relation of serum uric acid (SUA) with systemic inflammation has been little explored in humans and results have been inconsistent. We analyzed the association between SUA and circulating levels of interleukin-6 (IL-6), interleukin-1β (IL-1β), tumor necrosis factor- α (TNF-α) and C-reactive protein (CRP).

**Methods and Findings:**

This cross-sectional population-based study conducted in Lausanne, Switzerland, included 6085 participants aged 35 to 75 years. SUA was measured using uricase-PAP method. Plasma TNF-α, IL-1β and IL-6 were measured by a multiplexed particle-based flow cytometric assay and hs-CRP by an immunometric assay. The median levels of SUA, IL-6, TNF-α, CRP and IL-1β were 355 µmol/L, 1.46 pg/mL, 3.04 pg/mL, 1.2 mg/L and 0.34 pg/mL in men and 262 µmol/L, 1.21 pg/mL, 2.74 pg/mL, 1.3 mg/L and 0.45 pg/mL in women, respectively. SUA correlated positively with IL-6, TNF-α and CRP and negatively with IL-1β (Spearman r: 0.04, 0.07, 0.20 and 0.05 in men, and 0.09, 0.13, 0.30 and 0.07 in women, respectively, P<0.05). In multivariable analyses, SUA was associated positively with CRP (β coefficient ± SE = 0.35±0.02, P<0.001), TNF-α (0.08±0.02, P<0.001) and IL-6 (0.10±0.03, P<0.001), and negatively with IL-1β (−0.07±0.03, P = 0.027). Upon further adjustment for body mass index, these associations were substantially attenuated.

**Conclusions:**

SUA was associated positively with IL-6, CRP and TNF-α and negatively with IL-1β, particularly in women. These results suggest that uric acid contributes to systemic inflammation in humans and are in line with experimental data showing that uric acid triggers sterile inflammation.

## Introduction

A high level of serum uric acid (SUA) was found to predict the development of hypertension [1], [2], obesity [3], [4], insulin resistance [3], [5], kidney disease [2] and cardiovascular events [2], [6]. A potential mechanism by which uric acid (this encompasses SUA and the extravascular effects of the urate molecule) could be associated with cardiovascular morbidity is via inflammation [7], [8]. Experimental studies have demonstrated that tissue damage releases endogenous substances including uric acid which signals danger and stimulates inflammation [9]. SUA has significant effect on vascular smooth muscle cells. It has been shown that SUA when entering the vascular smooth muscle cell stimulates the release of C-reactive protein (CRP) and chemokine monocyte chemoattractant protein-1 (MCP-1), known to have a major role in the initiation of atherosclerotic lesions [10]–[12]. Uric acid also stimulates human mononuclear cells to produce interleukin 1β (IL-1β), interleukin 6 (IL-6) and tumor necrosis factor α (TNF-α) [2].

Few studies have investigated the relationship between SUA and systemic inflammation in humans. In small-sized studies including patients with chronic heart failure, SUA was associated positively with TNF- α [13], [14] and IL-6 [13]. In two population-based studies including 566 [15] and 1703 [16] healthy people, SUA was associated positively with CRP. SUA was positively associated with IL-6, TNF- α and CRP in 957 elderly people in Italy [17] and SUA predicted CRP increase during follow-up [18]. By contrast, SUA was not associated with IL-6, TNF- α and CRP in 333 elderly men in Taiwan [19].

Given the paucity of data from large-scale population-based studies and the inconsistent results obtained so far, we explored the associations of SUA with circulating levels of IL-6, IL-1β, TNF-α and CRP and assessed whether sex modified these associations in the CoLaus study.

## Methods

### Study population

The Colaus study is a cross-sectional population-based study conducted in Lausanne, Switzerland. Details of the study have been previously described [20]. Briefly, a simple, non-stratified random sampling of 19,830 participants, corresponding to 35% of the source population, was drawn, of which eventually 6184 participants were included. Inclusion criteria included a written informed consent, age between 35–75 years and being of Caucasian origin. The study was approved by Ethics Committee of the University of Lausanne. Recruitment began in June 2003 and ended in May 2006.

### Study procedure and measurements

Participants attended the outpatient clinic at the Centre Hospitalier Universitaire Vaudois (CHUV) in the morning after an overnight fast. They were asked to continue taking their medications as usual. This examination included a detailed health questionnaire, physical examination with anthropometric measures by trained and certified field interviewers and laboratory testing. In this analysis, smoking was defined as present if the participant reported to be current smoker at the time of examination and alcohol consumption was defined as present for participants who reported drinking alcohol at least once a day. Diuretic use was assessed by recording all the prescribed drugs taken by the participants and was considered as present if participants were using drugs belonging to any class of diuretics. Body mass index (BMI) was defined as weight divided by height in meter squared. Overweight was defined as BMI equal to or more than 25 kg/m^2^ and obesity as BMI greater than 30 kg/m^2^. Blood pressure was measured three times on the left arm using a clinically validated automatic oscillometric device (Omron HEM-907, Matsusaka, Japan) after applying the appropriate cuff size and after a period of 10 minutes rest with the subject in the sitting position. The average of the second and third values was used for analysis. Hypertension was defined as mean systolic blood pressure of ≥140 mmHg or mean diastolic blood pressure of ≥90 mmHg or presence of anti-hypertensive medication. A diagnosis of diabetes was made if fasting plasma glucose was greater than or equal to 7.0 mmol/l or presence of oral hypoglycaemic or insulin treatment.

Venous blood samples were collected after an overnight fasting. Most clinical assays were performed by the CHUV Clinical Laboratory on fresh blood samples whereas Pathway Diagnostics (Los Angeles, CA) measured insulin. Glucose was measured by glucose dehydrogenase (2.1% - 1.0% maximum inter and intra-batch coefficients of variation); serum and urinary creatinine by Jaffe kinetic compensated method (2.9% - 0.7%) and uric acid by uricase-PAP (1.0% - 0.5%). Glomerular filtration rate (GFR) was estimated using the abbreviated Modification of the Diet in Renal Disease (MDRD) formula: 186×(serum creatinine [µmol/L]/88.4) ^(−1.154)^×age ^(−0.203)^×F, where F = 1 for men and F = 0.742 for women [21].

Cytokine levels were measured using a multiplexed particle-based flow cytometric cytokine assay [22], a methodology used in other studies [23]. Milliplex kits were purchased from Millipore (Zug, Switzerland). The procedures closely followed the manufacturer's instructions. The analysis was conducted using a conventional flow cytometer (FC500 MPL, Beckman Coulter, Nyon, Switzerland). Lower detection limits for IL-1β, IL-6 and TNF-α were 0.2 pg/ml. Intra and inter-assay coefficients of variation were 15% and 16.7% for IL-1β, 16.9% and 16.1% for Il-6 and 12.5% and 13.5% for TNF-α, respectively. For quality control, repeated measurements were conducted for 80 subjects randomly drawn from the initial sample. High sensitive CRP (CRP) was assessed by immunoassay and latex HS (IMMULITE 1000–High, Diagnostic Products Corporation, LA, CA, USA) with maximum intra- and interbatch coefficients of variation of 1.3% and 4.6%, respectively.

### Statistical analysis

Because SUA levels strongly differ by sex, men and women were analyzed separately. Continuous variables were summarized as mean± standard deviation (SD) or as median and interquartile range [IQR] while categorical variables as number of subjects and percentages. We used t-test or Wilcoxon ranksum test and chi-square test to compare the differences in distribution of continuous and categorical covariates, respectively, between men and women. All values of IL-1β, IL-6 and TNF-α below the detection level (0.2 pg/ml), were substituted with a value (0.133) equivalent to two-thirds of the lower detection limit as recommended by Hornung et al [24] . Spearman's rank correlation test was used to analyze the association of SUA with inflammatory cytokines and Fischer's Z transformation to compare the correlation coefficients between men and women. We plotted the distribution of the inflammatory cytokines across sex-specific SUA quintiles and used a non-parametric test to assess for trends across the sex-specific SUA quintiles. These quintiles were generated separately in men and women, which leads to an equal proportion of men and women across quintiles. The distribution of unadjusted and adjusted beta-coefficients of log-transformed inflammatory markers across sex-specific SUA quintiles was performed using a linear regression. The P-value for trend was obtained by a test of ‘departure from linear trend’ using a likelihood ratio test (LRT) comparing a model assuming a linear trend for SUA with another estimating separate effects for each SUA quintiles. We considered the P-value obtained from a linear model as the value for P-trend whenever the LRT showed no difference in the fit of the two models. In the adjusted models, we controlled for only those co-variates that were both associated with SUA and inflammatory markers in our data; hence age, sex, BMI, alcohol intake, smoking, GFR, diabetes, hypertension and use of diuretics were included.

We examined the association of SUA (independent variable of interest) with log-transformed values of inflammatory markers as the dependant variable, one at a time using linear regression models. We started by univariate analysis and subsequently fitted models adjusting for (1) age, (2) age and BMI, (3) age, BMI, alcohol intake, smoking, GFR, diabetes, hypertension and use of diuretics. To assess if the relation was modified by gender, we included a multiplicative interaction parameter between sex and SUA into the linear models. We also performed the univariate and multivariable analysis after excluding values that were below the lower detection limit. We conducted sensitivity analysis excluding: (1) diabetic subjects (n = 401) (2) hypertensive subjects (n = 2223), (3) subjects treated with drugs that potentially influence SUA levels (including acetylsalicylic acid, diuretics, angiotensin converting enzymes inhibitors , angiotensin receptor blockers and other drugs known to induce hyperuricemia and hypouricemia) (n = 1168) and (4) subjects with cardiovascular diseases (CVD) (n = 246). We also conducted stratified analyses by overweight status and alcohol consumption (non-drinkers vs regular alcohol drinkers). The significance level used for two-sided tests was P<0.05. All tests were performed using Stata 11.0 (StataCorp, College Station, TX, USA).

## Results

Among the 6184 participants, 47% were men. The proportion of missing data ranged from 0.03% to 2% (for interleukins and TNF-α). The proportion of values that were below the lower limit of detection of the laboratory assays was 37.5%, 7.3% and 0.6% for IL-1β, IL-6 and TNF-α, respectively.

The main demographic and clinical characteristics of the Colaus population according to sex are presented in **Table 1**. SUA was significantly higher in men (361±75.7) than in women (270.6±67.2) as well as the prevalences of reported alcohol consumption and smoking. Men had higher BMI and fasting plasma glucose than women. The overall prevalences of diabetes and hypertension in the study population were 6% and 36%, respectively, with higher prevalences in men. A small proportion of participants (2.3%) were on diuretics and 16.6% were on other drugs (acetylsalicylic acid, ACE inhibitors, allopurinol and angiotensin receptor blockers) known to potentially influence SUA levels.

**Table 1:** Characteristics of the Colaus sample.

	Overall (n = 6,184)		Male (n = 2,933)		Female (n = 3251)		
	Mean	SD/IQR	Mean	SD/IQR	Mean	SD/IQR	p-value
**Age (years)**	53.1	10.8	52.6	10.8	53.5	10.7	<0.001
**Alcohol consumption, %**	25.4		36.1		15.7		<0.001
**Current smoking, %**	27		29.3		25		<0.001
**Diabetes, %**	6.5		9.6		3.7		<0.001
**Hypertension, %**	35.9		42.1		30.4		<0.001
**Diuretics use, %**	2.3		1.7		2.8		0.003
**BMI (kg/m^2^)**	25.8	4.6	26.6	4	25.1	4.9	<0.001
**Serum uric acid (µmol/L)**	313.5	84.5	361.1	75.7	270.6	67.2	<0.001
**Fasting glucose (mmol/l)**	5.6	1.1	5.8	1.2	5.3	1	<0.001
**GFR (ml/min/1.73 m^2^)**	83.6	16.6	86.7	17.4	80.7	15.2	<0.001
IL-1β (pg/mL)*	0.4	0.1–1.7	1.5	0.7–3.5	1.2	0.5–2.9	<0.001
IL-6(pg/mL)*	1.3	0.6–3.2	0.3	0.1–1.5	0.5	0.1–1.9	<0.001
TNF-α (pg/mL)*	2.9	1.8–4.5	3	1.9–4.6	2.7	1.7–4.4	<0.001
CRP (mg/L)*	1.3	0.6–2.7	1.2	0.6–2.6	1.3	0.6–2.9	0.013

BMI = body mass index; GFR = glomerular filtration rate (calculated according to Modification in Diet in Renal Disease equation); IL-1β = interleukin-1β; IL-6 = interleukin-6: TNF-α = tumour necrosis factor-alpha; CRP = ultrasensitive C-reactive protein.

Results are presented as mean (SD), percentages or median (interquartile range) for those marked with an asterix. Between-group comparisons by ttest, Chi-square test or Wilcoxon ranksum test.

Of the initial 6184 participants, 6085 (98.4%) had serum cytokines assessed. **Table 2** describes the correlations of the different inflammatory cytokines with SUA separately for men and women. With the exception of IL-1β which had an inverse association with SUA, all markers showed a significant positive correlation with SUA. The strongest correlation was observed between SUA and CRP both in men (r = 0.20, p<0.001) and women (r = 0.30, p<0.001). These correlations were significantly stronger in women than in men for IL-6, TNF-α and CRP (p-value for test of equality of the correlation coefficients being 0.032, 0.015 and <0.001 respectively).

**Table 2:** Spearman's correlation coefficient of inflammatory markers with uric acid according to sex.

	Male		Female		
	r	p-value	r	p-value	P-value*
IL-1β	−0.05	0.014	−0.07	<0.001	0.286
IL-6	0.04	0.036	0.09	<0.001	0.032
TNF-α	0.07	<0.001	0.13	<0.001	0.015
CRP	0.20	<0.001	0.30	<0.001	0.000

IL-1β = interleukin-1β; IL-6 = interleukin-6: TNF-α = tumour necrosis factor-alpha; CRP = ultrasensitive C-reactive protein.

*P-value testing the difference in correlation coefficient between men and women.

**Figure 1:**
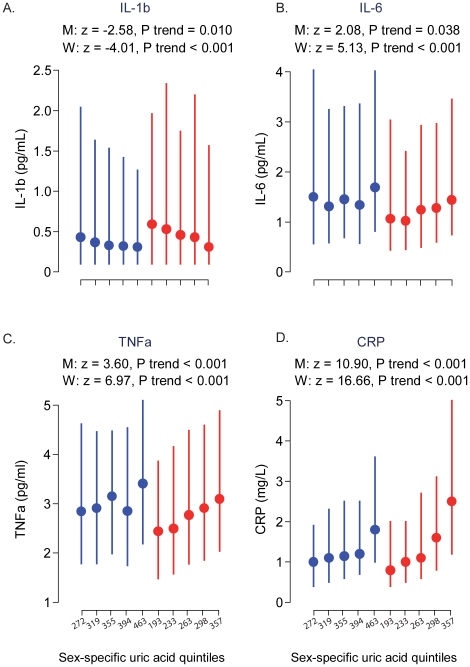
Distribution of inflammatory markers across sex-specific quintiles of serum uric acid by sex. Dots are median and bars are interquartile range. Blue colour indicates men and red colour indicates women. Z and p values represent non-parametric test for trends across quintiles. IL-1β = interleukin-1β; IL-6 = interleukin-6: TNF-α = tumour necrosis factor-alpha; CRP = ultrasensitive C-reactive protein; M = men; W = women.

**Figure 1** shows the median and the interquartile range of the different inflammatory cytokines plotted across the sex-specific quintiles of SUA separately in men and women. There was a significant negative trend for IL-1β with the relationship being stronger in women than in men (P-trend<0.001 in women vs. P-trend = 0.010 in men); women also had higher median values in almost all the quintile groups. Although IL-6 was showing a positive trend, the relationship was not as obvious in men. Strong significant positive trends (p<0.001) were observed for TNF-α and CRP both in men and women. These results are also supported by findings in **Table 3** and **4** which present the beta coefficients using the inflammatory cytokines as dependent and sex-specific SUA quintiles as independent variable. On adjusting for important co-variates, only CRP still showed strong positive trends in men and women.

**Table 3:** Unadjusted and adjusted linear regression coefficients of sex-specific quintiles of uric acid (95% CI) on log of inflammatory markers in males.

		Q1	Q2	Q3	Q4	Q5	P-trend
Serum uric acid (µmol/L)		271.5	319	355	394	463	
**IL-1β (pg/mL)**	**Unadjusted**	Ref	−0.07	−0.12	−0.18	−0.24	0.008
			(−0.27;0.11)	(−0.31;0.07)	(−0.37;0.01)	(−0.43;−0.05)	
	**Fully adjusted**	Ref	−0.08	−0.09	−0.14	−0.13	0.182
			(−0.27;0.11)	(−0.28;0.09)	(−0.33;0.06)	(−0.33;0.08)	
	**Fully adjusted excluding BMI**	Ref	−0.07	−0.09	−0.12	−0.10	0.261
			(−0.26;0.12)	(−0.27;0.10)	(−0.31;0.07)	(−0.30;0.10)	
**IL-6(pg/mL)**	**Unadjusted**	Ref	−0.13	0.04	−0.11	0.14	0.121
			(−0.30;0.03)	(−0.12;0.20)	(−0.28;0.05)	(−0.03;0.30)	
	**Fully adjusted**	Ref	−0.15	0.01	−0.15	0.06	0.603
			(−0.32;0.01)	(−0.15;0.18)	(−0.32;0.02)	(−0.12;0.23)	
	**Fully adjusted excluding BMI**	Ref	−0.13	0.04	−0.11	0.13	0.164
			(−0.30;0.03)	(−0.12;0.20)	(−0.27;0.06)	(−0.04;0.30)	
**TNF-α (pg/mL)**	**Unadjusted**	Ref	−0.01	0.07	−0.01	0.18	0.002
			(−0.11;0.09)	(−0.03;0.17)	(−0.11;0.10)	(0.08;0.28)	
	**Fully adjusted**	Ref	−0.02	0.05	−0.04	0.12	0.088
			(−0.13;0.08)	(−0.05;0.15)	(−0.15;0.06)	(0.01;0.23)	
	**Fully adjusted excluding BMI**	Ref	−0.02	0.06	−0.02	0.15	0.014
			(−0.12;0.09)	(−0.04;0.16)	(−0.12;0.08)	(0.05;0.26)	
**CRP (mg/L)**	**Unadjusted**	Ref	0.21	0.28	0.35	0.66	<0.001
			(0.09;0.33)	(0.16;0.39)	(0.23;0.47)	(0.55;0.78)	
	**Fully adjusted**	Ref	0.15	0.20	0.22	0.41	<0.001
			(0.04;0.26)	(0.09;0.31)	(0.11;0.34)	(0.29;0.53)	
	**Fully adjusted excluding BMI**	Ref	0.21	0.28	0.36	0.62	<0.001
			(0.09;0.32)	(0.17;0.40)	(0.24;0.48)	(0.50;0.74)	

*median value of serum uric acid in each quintile. IL-1β = interleukin-1β; IL-6 = interleukin-6: TNF-α = tumour necrosis factor-alpha; CRP = ultrasensitive C-reactive protein.

Adjusted for age, alcohol intake, smoking, BMI, GFR (calculated according to Modification in Diet in Renal Disease equation), diabetes, hypertension & use of diuretics.

**Table 4:** Unadjusted and adjusted linear regression coefficients of sex-specific quintiles of uric acid (95% CI) on log of inflammatory markers in females.

		Q1	Q2	Q3	Q4	Q5	P-trend
Serum uric acid (µmol/L)		193	233	263	298	357	
**IL-1β (pg/mL)**	**Unadjusted**	Ref	−0.01	−0.15	−0.13	−0.34	<0.001
			(−0.19;0.17)	(−0.33;0.03)	(−0.31;0.05)	(−0.52;−0.16)	
	**Fully adjusted**	Ref	0.01	−0.09	−0.03	−0.18	0.106
			(−0.17;0.19)	(−0.27;0.10)	(−0.21;0.16)	(−0.39;0.02)	
	**Fully adjusted excluding BMI**	Ref	0.01	−0.09	−0.04	−0.21	0.050
			(−0.17;0.19)	(−0.27;0.09)	(−0.22;0.15)	(−0.40;−0.01)	
**IL-6(pg/mL)**	**Unadjusted**	Ref	−0.10	0.08	0.11	0.28	<0.001
			(−0.26;0.06)	(−0.08;0.25)	(−0.05;0.27)	(0.11;0.44)	
	**Fully adjusted**	Ref	−0.11	0.03	0.02	0.08	0.179
			(−0.28;0.05)	(−0.13;0.20)	(−0.15;0.19)	(−0.10;0.27)	
	**Fully adjusted excluding BMI**	Ref	−0.10	0.07	0.10	0.22	0.002
			−0.26;0.06)	(−0.09;0.24)	(−0.07;0.27)	(0.04;0.39)	
**TNF-α (pg/mL)**	**Unadjusted**	Ref	0.06	0.14	0.19	0.26	<0.001
			(−0.04;0.16)	(0.04;0.24)	(0.08;0.29)	(0.16;0.36)	
	**Fully adjusted**	Ref	0.04	0.08	0.09	0.09	0.065
			(−0.06;0.14)	(−0.02;0.18)	(−0.01;0.20)	(−0.02;0.21)	
	**Fully adjusted excluding BMI**	Ref	0.04	0.10	0.13	0.16	0.001
			(−0.06;0.14)	(0.00;0.20)	(0.03;0.23)	(0.05;0.27)	
**CRP (mg/L)**	**Unadjusted**	Ref	0.07	0.30	0.52	0.92	<0.001
			(−0.04;0.19)	(0.18;0.42)	(0.40;0.64)	(0.80;1.04)	
	**Fully adjusted**	Ref	0.04	0.15	0.23	0.39	<0.001
			(−0.07;0.15)	(0.04;0.26)	(0.11;0.34)	(0.27;0.51)	
	**Fully adjusted excluding BMI**	Ref	0.08	0.27	0.48	0.80	<0.001
			(−0.03;0.20)	(0.16;0.39)	(0.36;0.60)	(0.67;0.93)	

*median value of serum uric acid in each quintile. IL-1β = interleukin-1β; IL-6 = interleukin-6: TNF-α = tumour necrosis factor-alpha; CRP = ultrasensitive C-reactive protein.

Adjusted for age, alcohol intake, smoking, BMI, GFR (calculated according to Modification in Diet in Renal Disease equation), diabetes, hypertension & use of diuretics.

In the univariate analysis (**Table 5**), SUA was a strong significant predictor of IL-1β, IL-6, TNF-α and CRP in women; conversely, in men a strong significant association was evident only for TNF-α and CRP. The beta coefficients for SUA were almost twice as large in women as in men. Adjustment for age slightly attenuated all the coefficients, whereas adding BMI as a covariate in the multivariable models substantially reduced the size of the beta coefficients for SUA, particularly in women and this reduction was massive for CRP in both men and women. The associations, however, still remained significant except for IL-6. Further adjustment for alcohol intake, smoking, GFR, diabetes, hypertension and use of diuretics, had little impact on the associations. Removing values that were below the lower detection limits led to very similar results. Similarly, sensitivity analysis excluding diabetics, subjects on diuretics or with cardiovascular disease did not substantially change the results and led to similar conclusions. However, on excluding hypertensive subjects, SUA was no longer associated with IL-6.

**Table 5:** Linear Regression of serum uric acid (per 100 µmol/L) on log-transformed inflammatory markers, overall and by sex.

	Overall			Male			Female			P-value*
Univariate	β-coeff	S.E	P-value	β-coeff	S.E	P-value	β-coeff	S.E	P-value	
IL-1β (pg/mL)	−0.14	0.03	<0.001	−0.10	0.04	0.015	−0.19	0.04	<0.001	0.061
IL-6 (pg/mL)	0.14	0.02	<0.001	0.07	0.04	0.040	0.18	0.04	<0.001	0.076
TNF-α (pg/mL)	0.1	0.01	<0.001	0.08	0.02	<0.001	0.15	0.02	<0.001	0.030
CRP (mg/L)	0.26	0.02	<0.001	0.30	0.03	<0.001	0.51	0.03	<0.001	<0.001
**Age & sex adjusted**										
IL-1β (pg/mL)	−0.09	0.03	0.004	−0.06	0.04	0.120	−0.12	0.05	0.009	0.269
IL-6 (pg/mL)	0.1	0.03	<0.001	0.05	0.04	0.161	0.17	0.04	<0.001	0.156
TNF-α (pg/mL)	0.09	0.02	<0.001	0.06	0.02	0.003	0.11	0.03	<0.001	0.128
CRP (mg/L)	0.34	0.02	<0.001	0.26	0.03	<0.001	0.46	0.03	<0.001	<0.001
**Age, sex & BMI adjusted**										
IL-1β (pg/mL)	−0.08	0.03	0.013	−0.06	0.04	0.141	−0.11	0.05	0.028	0.288
IL-6 (pg/mL)	0.04	0.03	0.128	0.02	0.04	0.625	0.09	0.04	0.042	0.387
TNF-α (pg/mL)	0.06	0.02	<0.001	0.05	0.02	0.040	0.08	0.03	0.004	0.278
CRP (mg/L)	0.17	0.02	<0.001	0.16	0.03	<0.001	0.21	0.03	<0.001	0.046
**Fully adjusted** **										
IL-1β (pg/mL)	−0.07	0.03	0.034	−0.04	0.04	0.333	−0.11	0.05	0.030	0.218
IL-6 (pg/mL)	0.05	0.03	0.107	0.04	0.04	0.324	0.07	0.05	0.110	0.399
TNF-α (pg/mL)	0.06	0.02	0.003	0.05	0.02	0.045	0.06	0.03	0.025	0.297
CRP (mg/L)	0.19	0.02	<0.001	0.19	0.03	<0.001	0.22	0.03	<0.001	0.047
**Fully adjusted excluding BMI**										
IL-1β (pg/mL)	−0.07	0.03	0.027	−0.03	0.04	0.445	−0.12	0.05	0.012	0.418
IL-6 (pg/mL)	0.10	0.03	<0.001	0.07	0.04	0.063	0.15	0.04	0.001	0.112
TNF-α(pg/mL)	0.08	0.02	<0.001	0.06	0.02	0.006	0.10	0.03	<0.001	0.118
CRP (mg/L)	0.35	0.02	<0.001	0.29	0.03	<0.001	0.44	0.03	<0.001	<0.001

*P-value for interaction between serum uric acid and sex. IL-1β = interleukin-1β; IL-6 = interleukin-6: TNF-α = tumour necrosis factor-alpha; CRP = ultrasensitive C-reactive protein.

**Adjusted for age, sex, alcohol intake, smoking, BMI, GFR (calculated according to Modification in Diet in Renal Disease equation), diabetes, hypertension & use of diuretic.

The associations of SUA with log-transformed inflammatory cytokines were also present in normal-weight men and women for TNF-α and CRP, but were not significant for IL-6 and IL-1β (**Table S1**). In teetotalers, SUA was associated with all cytokines in women but only with CRP in men (**Table S1**). These results show that the reported associations between SUA and inflammatory markers are not due to alcohol intake.

## Discussion

In this population-based study of Caucasians aged 35 to 75 years, we found a strong positive association of SUA with CRP and a weaker, albeit significant, positive association of SUA with TNF-α and IL-6 in men and women, which was in part mediated by BMI. These findings support the hypothesis that uric acid is involved in sterile (i.e., non-infectious) inflammation by triggering the release of inflammatory cytokines, in particular CRP and TNF-α. Such systemic inflammation may eventually contribute to the development of atherosclerosis, hypertension and diabetes. These findings are in line with recent experimental data in mice showing that uric acid represents a major proinflammatory damage-associated molecular pattern (DAMP) [25].

To the best of our knowledge, this is the largest population-based study to assess the relationship between SUA and circulatory inflammatory cytokines. As compared to other population-based studies such as the InCHIANTI study [17], the Colaus population had lower mean age and fewer participants suffered from diabetes, hypertension or cardiovascular diseases. Hence, this study provides information on the relationship between SUA and the different inflammatory cytokines in relatively young and healthy individuals.

As in the InCHIANTI study [17], the findings from the present study showed that SUA was positively associated with CRP. Similar findings were also reported by Frohlich et al [16] and Saito et al [15]. Indeed, in the present study, CRP remained strongly significant even after adjustment for important potential confounders such as BMI. Although controversial, these finding are in keeping with the theory that high uric acid may contribute to the atherosclerotic process by stimulating the release of CRP, an established marker of inflammation. In fact, *in vitro* studies support this hypothesis; it has been shown that uric acid enters the vascular smooth muscle cells, where it stimulates pro-inflammatory response, leading to increased cell proliferation and production of CRP and other inflammatory mediators [10], [26].

SUA was positively and independently associated with TNF-α in both men and women in the current study, confirming previous findings from patients with chronic heart failure [13] and from the InCHIANTI study [17]. These findings are consistent with experimental data supporting the role of SUA in stimulating the release of TNF-α. A marked increase in circulating TNF-α levels has been observed on infusion of uric acid into mice [27]. More recently, cell culture experiments by Bordoni et al [28] showed the role of uric acid in signal transduction in the apoptotic pathway, subsequently leading to inflammatory reaction. They also demonstrated that uric acid stimulates the mononuclear cells to produce TNF-α [28].

IL-6 is a major stimulus for production of most acute phase proteins and plays an important role as a mediator of inflammation [29]. In this study, we observed a positive and significant association between SUA and IL-6 as long as it was not adjusted for BMI, indicating that this relationship appears to be largely dependent on BMI. The finding of a positive association between SUA and IL-6 is also consistent with the finding from patients with chronic heart failure [13] and from the InCHIANTI study [17], but contrasts with a study on institutionalized elderly men in Taiwan [19]. The positive findings are in agreement with the finding that uric acid stimulates human mononuclear cells to produce IL-6 [2].

A significant negative association of SUA with circulating IL-1β levels was found in men and women. To the best of our knowledge, the only study in human that assessed the relationships between SUA and IL-1β found no association between the two [13]; however, the sample size was limited to 39 patients with chronic heart failure and 16 healthy controls. Considering the key role of the IL-1 pathway in sterile inflammation with IL-1β acting as a potent proinflammatory cytokine [30], the observation that monosodium urate crystals stimulate IL-1 production by neutrophils [31], and the recent identification of uric acid as a proinflammatory DAMP [25], the negative association of SUA with IL-1β was unexpected and its interpretation is not straightforward. First, the results of *in vitro* and *in vivo* experimental data do not always translate to humans. This is, for instance, true for SUA that is substantially higher in humans than in mice because uricase is inactive in humans. Second, small amounts of IL-1β are known to be sufficient to cause biological effects [30] and current IL-1β assays may not be sensitive enough to capture the whole spectrum of biologically relevant effects. Third, it is not clear that circulating IL-1β levels are a good proxy for local IL-1β activity in specific organs. There is also a feedback control on IL-1β levels in chronic inflammation and what we are seeing is a chronic state and not an acute reaction.

In addition, these results should be interpreted with caution because, in the present study, 38% of the participants had IL-1β below detection levels. Nevertheless, this value is, not unusual and actually lower than reported elsewhere [32], [33]. Interestingly, analyses conducted after removing subjects with values below the detection limit yielded very similar results. In the other study reporting data on circulating IL-1β levels in humans, the issue of undetectable values was not discussed [13], which limits comparison. More sensitive assays are needed to better explore and interpret the association of circulating IL-1β with SUA and related cardiovascular traits.

Of interest, in the current study, the association between SUA and inflammatory cytokines appeared to depend on BMI. It was the addition of BMI into the models, and not so much of the other co-variates, that attenuated the effect sizes. Obesity is associated with increased SUA levels [3], [34] and overproduction of inflammatory molecules like TNF- α and IL-6 by the white adipose tissue [35]. Yet, the nature, and direction, of the causal link between hyperuricemia and obesity is unclear. Masuo et al showed that SUA predicts subsequent weight gain in nonobese, healthy young men [36]. This is further substantiated by experimental studies in animals which showed that allopurinol prevents both fructose-induced hyperuricaemia and weight gain [37]. Although these results could suggest that hyperuricemia might cause obesity, one cannot differentiate, based on these results, whether (1) hyperuricemia directly causes obesity or (2) hyperuricemia and obesity share a common cause (e.g. fructose intake). Hence, BMI may lie in the causal pathway linking SUA to inflammatory markers and controlling for it may actually represent an overadjustment, thus explaining the attenuation of the effect sizes in the current study.

The strengths of this study are its population-based design, large sample size, the availability of detailed information on major confounders and the high quality of cytokine-dosing, resulting in good reproducibility of results. The potential limitation is the cross-sectional nature of the study which does not allow us to infer causality and the relatively high number of undetectable values for IL-1β, which may bias the results and limit the interpretation of the results.

In conclusion, in this population-based sample, SUA was positively associated with CRP, TNF-α and IL-6 in both men and women suggesting that uric acid may have a role in inflammation and subsequent inflammatory related diseases. The relation between SUA and IL-1β merits further investigation. Our findings may be clinically relevant in terms of primary prevention strategies for chronic disease which may necessitate the need to consider high SUA as a potential risk factor.

## Supporting Information

Table S1 Fully adjusted models (excluding BMI) of uric acid and log of inflammatory markers by BMI status and alcohol intake.
(DOC)

## References

[1] Alper AB, Chen W, Yau L, Srinivasan SR, Berenson GS (2005). Childhood uric acid predicts adult blood pressure: the Bogalusa
Heart Study.. Hypertension.

[2] Johnson RJ, Kang DH, Feig D, Kivlighn S, Kanellis J (2003). Is there a pathogenetic role for uric acid in hypertension and
cardiovascular and renal disease?. Hypertension.

[3] Bonora E, Targher G, Zenere MB, Saggiani F, Cacciatori V (1996). Relationship of uric acid concentration to cardiovascular risk
factors in young men. Role of obesity and central fat distribution. The
Verona Young Men Atherosclerosis Risk Factors Study.. Int J Obes Relat Metab Disord.

[4] Ogura T, Matsuura K, Matsumoto Y, Mimura Y, Kishida M (2004). Recent trends of hyperuricemia and obesity in Japanese male
adolescents, 1991 through 2002.. Metabolism.

[5] Nakanishi N, Okamoto M, Yoshida H, Matsuo Y, Suzuki K (2003). Serum uric acid and risk for development of hypertension and
impaired fasting glucose or Type II diabetes in Japanese male office
workers.. Eur J Epidemiol.

[6] Alderman MH, Cohen H, Madhavan S, Kivlighn S (1999). Serum uric acid and cardiovascular events in successfully treated
hypertensive patients.. Hypertension.

[7] Manzato E (2007). Uric acid: an old actor for a new role.. Intern Emerg Med.

[8] Montalcini T, Gorgone G, Gazzaruso C, Sesti G, Perticone F (2007). Relation between serum uric acid and carotid intima-media
thickness in healthy postmenopausal women.. Intern Emerg Med.

[9] Martinon F, Petrilli V, Mayor A, Tardivel A, Tschopp J (2006). Gout-associated uric acid crystals activate the NALP3
inflammasome.. Nature.

[10] Johnson RJ, Rodriguez-Iturbe B, Kang DH, Feig DI, Herrera-Acosta J (2005). A unifying pathway for essential hypertension.. Am J Hypertens.

[11] Kang DH, Nakagawa T, Feng L, Watanabe S, Han L (2002). A role for uric acid in the progression of renal
disease.. J Am Soc Nephrol.

[12] Rao GN, Corson MA, Berk BC (1991). Uric acid stimulates vascular smooth muscle cell proliferation by
increasing platelet-derived growth factor A-chain
expression.. J Biol Chem.

[13] Leyva F, Anker SD, Godsland IF, Teixeira M, Hellewell PG (1998). Uric acid in chronic heart failure: a marker of chronic
inflammation.. Eur Heart J.

[14] Olexa P, Olexova M, Gonsorcik J, Tkac I, Kisel'ova J (2002). Uric acid–a marker for systemic inflammatory response in
patients with congestive heart failure?. Wien Klin Wochenschr.

[15] Saito M, Ishimitsu T, Minami J, Ono H, Ohrui M (2003). Relations of plasma high-sensitivity C-reactive protein to
traditional cardiovascular risk factors.. Atherosclerosis.

[16] Frohlich M, Imhof A, Berg G, Hutchinson WL, Pepys MB (2000). Association between C-reactive protein and features of the
metabolic syndrome: a population-based study.. Diabetes Care.

[17] Ruggiero C, Cherubini A, Ble A, Bos AJ, Maggio M (2006). Uric acid and inflammatory markers.. Eur Heart J.

[18] Ruggiero C, Cherubini A, Miller E, Maggio M, Najjar SS (2007). Usefulness of uric acid to predict changes in C-reactive protein
and interleukin-6 in 3-year period in Italians aged 21 to 98
years.. Am J Cardiol.

[19] Chang CH, Chen YM, Chuang YW, Liao SC, Lin CS (2009). Relationship between hyperuricemia (HUC) and metabolic syndrome
(MS) in institutionalized elderly men.. Arch Gerontol Geriatr.

[20] Firmann M, Mayor V, Vidal PM, Bochud M, Pecoud A (2008). The CoLaus study: a population-based study to investigate the
epidemiology and genetic determinants of cardiovascular risk factors and
metabolic syndrome.. BMC Cardiovasc Disord.

[21] Levey AS, Stevens LA, Schmid CH, Zhang YL, Castro AF (2009). A new equation to estimate glomerular filtration
rate.. Ann Intern Med.

[22] Vignali DA (2000). Multiplexed particle-based flow cytometric
assays.. J Immunol Methods.

[23] von KR, Begre S, Abbas CC, Saner H, Gander ML (2010). Inflammatory biomarkers in patients with posttraumatic stress
disorder caused by myocardial infarction and the role of depressive
symptoms.. Neuroimmunomodulation.

[24] Hornung R, Reed L (1990). Estimation of average concentration in the presence of
nondectable values.. Appl Occup Environ Hyg.

[25] Kono H, Chen CJ, Ontiveros F, Rock KL (2010). Uric acid promotes an acute inflammatory response to sterile cell
death in mice.. J Clin Invest.

[26] Kanellis J, Watanabe S, Li JH, Kang DH, Li P (2003). Uric acid stimulates monocyte chemoattractant protein-1
production in vascular smooth muscle cells via mitogen-activated protein
kinase and cyclooxygenase-2.. Hypertension.

[27] Netea MG, Kullberg BJ, Blok WL, Netea RT, van der Meer JW (1997). The role of hyperuricemia in the increased cytokine production
after lipopolysaccharide challenge in neutropenic mice.. Blood.

[28] Bordoni V, De Cal M, Rassu M, Cazzavillan S, Segala C (2005). Protective effect of urate oxidase on uric acid induced-monocyte
apoptosis.. Curr Drug Discov Technol.

[29] Yudkin JS, Kumari M, Humphries SE, Mohamed-Ali V (2000). Inflammation, obesity, stress and coronary heart disease: is
interleukin-6 the link?. Atherosclerosis.

[30] Rock KL, Latz E, Ontiveros F, Kono H (2010). The sterile inflammatory response.. Annu Rev Immunol.

[31] Roberge CJ, de Medicis R, Dayer JM, Rola-Pleszczynski M, Naccache PH (1994). Crystal-induced neutrophil activation. V. Differential production
of biologically active IL-1 and IL-1 receptor antagonist.. J Immunol.

[32] Spranger J, Kroke A, Mohlig M, Hoffmann K, Bergmann MM (2003). Inflammatory cytokines and the risk to develop type 2 diabetes:
results of the prospective population-based European Prospective
Investigation into Cancer and Nutrition (EPIC)-Potsdam
Study.. Diabetes.

[33] Wong HL, Pfeiffer RM, Fears TR, Vermeulen R, Ji S (2008). Reproducibility and correlations of multiplex cytokine levels in
asymptomatic persons.. Cancer Epidemiol Biomarkers Prev.

[34] Matsuura F, Yamashita S, Nakamura T, Nishida M, Nozaki S (1998). Effect of visceral fat accumulation on uric acid metabolism in
male obese subjects: visceral fat obesity is linked more closely to
overproduction of uric acid than subcutaneous fat obesity.. Metabolism.

[35] Bastard JP, Maachi M, Lagathu C, Kim MJ, Caron M (2006). Recent advances in the relationship between obesity,
inflammation, and insulin resistance.. Eur Cytokine Netw.

[36] Masuo K, Kawaguchi H, Mikami H, Ogihara T, Tuck ML (2003). Serum uric acid and plasma norepinephrine concentrations predict
subsequent weight gain and blood pressure elevation.. Hypertension.

[37] Nakagawa T, Hu H, Zharikov S, Tuttle KR, Short RA (2006). A causal role for uric acid in fructose-induced metabolic
syndrome.. Am J Physiol Renal Physiol.

