# Supplementary material for: Elevated Serum Uric Acid Is Associated with High Circulating Inflammatory Cytokines in the Population-Based Colaus Study
Source: PLoS One. 2011 May 20;6(5):e19901. doi: 10.1371/journal.pone.0019901 (PMC3098830; doi:10.1371/journal.pone.0019901)
Supplement: Table S1 — Fully adjusted models (excluding BMI) of uric acid and log of inflammatory markers by BMI status and alcohol intake. (DOC) [file pone.0019901.s001.doc]

**Table S1: Fully adjusted (excluding BMI) linear regression** coefficient of uric acid (per 100 µmol/L) on log of inflammatory markers by BMI status and alcohol intake

|  | **Overall** | | | **Male** | | | **Female** | | | **P-value*** |
| --- | --- | --- | --- | --- | --- | --- | --- | --- | --- | --- |
|  | β-coeff | S.E | P-value | β-coeff | S.E | P-value | β-coeff | S.E | P-value |  |
| **Stratified by overweight status** | | |  |  |  |  |  |  |  |  |
| *BMI<25 kg/m^2^ (N=2922)* | |  |  | N=1085 |  |  | N=1837 |  |  |  |
| IL-1β(pg/mL) | -0.04 | 0.05 | 0.442 | 0.01 | 0.08 | 0.906 | -0.09 | 0.07 | 0.234 | 0.325 |
| IL-6 (pg/mL) | 0.08 | 0.05 | 0.133 | 0.04 | 0.07 | 0.548 | 0.11 | 0.07 | 0.122 | 0.593 |
| TNF-α (pg/mL) | 0.12 | 0.03 | <0.001 | 0.12 | 0.04 | 0.006 | 0.12 | 0.04 | 0.004 | 0.613 |
| CRP (mg/L) | 0.25 | 0.03 | <0.001 | 0.30 | 0.05 | <0.001 | 0.22 | 0.05 | <0.001 | 0.429 |
| *BMI>=25 kg/m^2^ (N=3159)* | |  |  | N=1795 |  |  | N=1364 |  |  |  |
| IL-1β(pg/mL) | -0.08 | 0.04 | 0.049 | -0.05 | 0.05 | 0.382 | -0.15 | 0.07 | 0.031 | 0.593 |
| IL-6 (pg/mL) | 0.09 | 0.04 | 0.008 | 0.08 | 0.04 | 0.087 | 0.14 | 0.06 | 0.022 | 0.305 |
| TNF-α (pg/mL) | 0.04 | 0.02 | 0.079 | 0.03 | 0.03 | 0.243 | 0.05 | 0.04 | 0.164 | 0.454 |
| CRP (mg/L) | 0.24 | 0.02 | <0.001 | 0.18 | 0.03 | <0.001 | 0.36 | 0.04 | <0.001 | 0.004 |
| **Stratified by alcohol consumption** | | |  |  |  |  |  |  |  |  |
| *Non-drinkers (N=4541)* | |  |  | N=1840 |  |  | N=2701 |  |  |  |
| IL-1β(pg/mL) | -0.07 | 0.04 | 0.070 | -0.01 | 0.06 | 0.819 | -0.13 | 0.05 | 0.016 | 0.377 |
| IL-6 (pg/mL) | 0.11 | 0.03 | 0.002 | 0.05 | 0.05 | 0.283 | 0.16 | 0.05 | 0.001 | 0.059 |
| TNF-α (pg/mL) | 0.08 | 0.02 | <0.001 | 0.05 | 0.03 | 0.066 | 0.11 | 0.03 | <0.001 | 0.110 |
| CRP (mg/L) | 0.39 | 0.02 | <0.001 | 0.30 | 0.03 | <0.001 | 0.47 | 0.04 | <0.001 | <0.001 |
| *Drinkers (N=1540)* | |  |  | N=1040 |  |  | N=500 |  |  |  |
| IL-1β(pg/mL) | -0.06 | 0.06 | 0.252 | -0.05 | 0.07 | 0.418 | -0.08 | 0.11 | 0.471 | 0.617 |
| IL-6 (pg/mL) | 0.09 | 0.05 | 0.059 | 0.10 | 0.06 | 0.073 | 0.08 | 0.10 | 0.415 | 0.972 |
| TNFα(pg/mL) | 0.08 | 0.03 | 0.018 | 0.08 | 0.04 | 0.029 | 0.06 | 0.07 | 0.341 | 0.947 |
| CRP (mg/L) | 0.29 | 0.04 | <0.001 | 0.27 | 0.04 | <0.001 | 0.33 | 0.07 | <0.001 | 0.064 |

*****P-value for interaction between serum uric acid and sex. IL-1β= interleukin-1β; IL-6=interleukin-6: TNF-α=tumour necrosis factor-alpha; CRP=ultrasensitive C-reactive protein

N corresponds to the lowest available sample

Adjusted for age, sex, alcohol intake, smoking, GFR (calculated according to Modification in Diet in Renal Disease equation), diabetes, hypertension & use of diuretics
